# Comparison of the effectiveness of a tailored cognitive behavioural therapy with a supportive listening intervention for depression in those newly diagnosed with multiple sclerosis (the ACTION-MS trial): protocol of an assessor-blinded, active comparator, randomised controlled trial

**DOI:** 10.1186/s13063-019-4018-8

**Published:** 2020-01-20

**Authors:** Litza Kiropoulos, Trevor Kilpatrick, Tomas Kalincek, Leonid Cherulov, Elizabeth McDonald, Tissa Wijeratne, Jennifer Threader, Vanja Rozenblat, Neil Simpson-O’Brien, Anneke Van Der Walt, Lisa Taylor

**Affiliations:** 1 Melbourne School of Psychological Sciences, University of Melbourne, Melbourne, Victoria 3010 Australia; 2 Department of Neurology, Royal Melbourne Hospital, Melbourne, Victoria Australia; 3 Florey Institute of Neuroscience and Mental Health, Melbourne, Victoria Australia; 4 Rehabilitation and Neuroimmunology, Department of Neuroscience, St Vincent’s Hospital, Melbourne, Victoria Australia; 5 Department of Neurology, Western Health, Sunshine, Victoria Australia; 6 Melbourne Dental School, University of Melbourne, Carlton, Victoria Australia; 7 Department of Neurosciences, Monash University, Melbourne, Victoria Australia

**Keywords:** Depression, Cognitive behavioural therapy (CBT), Multiple sclerosis, Newly diagnosed, Randomised controlled trial, Anxiety

## Abstract

**Background:**

Multiple sclerosis (MS) is an unpredictable, chronic neurological disease accompanied with high rates of depression and anxiety, particularly in the early stages of diagnosis. There is evidence to suggest that cognitive behavioural therapy (CBT) is effective for the treatment of depression amongst individuals with MS; however, there is a paucity of tailored CBT interventions designed to be offered in the newly diagnosed period. This trial is the first to assess the effectiveness and cost-effectiveness of a tailored CBT intervention compared to a supportive listening (SL) intervention amongst individuals with MS who are depressed.

**Methods:**

ACTION-MS is a two-arm parallel group, assessor-blinded, active comparator, randomised controlled trial which will test whether a tailored CBT-based intervention compared to an SL intervention can reduce depression and related factors such as anxiety, fatigue, pain and sleep problems in those newly diagnosed with MS. Sixty participants who are within 5 years of having received a diagnosis of MS and scored within the mild to moderate range of depression on the Beck Depression Inventory (BDI-II) will be recruited from MS clinics located across three hospital sites in Melbourne, Australia. The primary outcome is depression severity using the BDI-II at post-assessment. Intervention satisfaction and acceptability will be assessed. A cost-effectiveness analysis will also be conducted. Data will be analysed on an intention-to-treat basis.

**Discussion:**

There is a scarcity of psychological interventions for depression targeting the newly diagnosed period. However, interventions during this time point have the potential to have a major impact on the mental and physical wellbeing of those newly diagnosed with MS. The current trial will provide data on the effectiveness of a tailored CBT intervention for the treatment of depression in those newly diagnosed with MS. Findings will also provide effect size estimates that can be used to power a later-stage multi-centre trial of treatment efficacy, and will provide information on the mechanisms underlying any treatment effects and cost-effectiveness data for delivering this intervention in outpatient MS clinics.

**Trial registration:**

ISRCTN trials registry, ISRCTN63987586. Current controlled trials. Retrospectively registered on 20 October 2017.

## Background

High levels of depression and anxiety have been found around the time of multiple sclerosis (MS) diagnosis [1, 2] with up to 36% of individuals with MS reporting high rates of depression and anxiety in the first 2 years of diagnosis [2–4]. Point prevalence rates of depression range from 10 to 42%, with up to 50% of individuals with MS experiencing depression in their lifetime [5, 6], which is higher than the value for any other neurological condition or chronic illness [7, 8]. Risk factors for depression amongst individuals with MS include functional status, anxiety, fatigue, cognitive impairment, pain [5, 9], losses in physical and cognitive function, occupational roles and social support [9], a reduction in previously pursued activities [10] and disease characteristics including MS illness duration, symptom severity, neurological disability and disease status [7, 11]. Demyelination and inflammation in certain parts of the brain and associated neurological changes have also been found to play a role in the development of depressive symptoms [5]. Depression in individuals with MS has been associated with poor psycho-social and treatment outcomes such as anxiety, fatigue, pain, sleep disturbances, poorer quality of life [2, 12–14], restricted social and economic opportunities [15], reduced medication adherence [16] and MS symptom exacerbations [17].

There is mounting evidence for the effectiveness of cognitive behavioural therapy (CBT) in treating depression amongst individuals with MS. In a recent systematic review and meta-analysis looking at interventions of depression in individuals with MS, Fiest and colleagues [18] identified eight randomised controlled trials that employed CBT-based interventions for the treatment of depression in those with MS and found that the severity of depression scores improved in all trials (standardised mean difference [SMD] – 0.45; 95% confidence interval [CI] − 0.74, − 0.16). Despite these promising findings, research in the treatment of depression amongst individuals with MS has been limited in its methodology, with a paucity of interventions targeted towards individuals who are newly diagnosed. Early psychological intervention is important, considering rates of depression and anxiety have been found to be at clinically high levels around the time of diagnosis [1–4]. Furthermore, if depression is left untreated, it will worsen and contribute to further deterioration, having an impact on the course of MS [19], resulting in exacerbation of relapses [17], higher suicide rates [20] and lower treatment adherence [21]. Early provision of CBT may increase psychological wellbeing, lead to improvements in treatment thereby altering MS disease progression and also lead to better social and functional outcomes for individuals with MS [2].

Tailored CBT approaches adapted specifically for individuals with MS have been found to be more effective and preferred over generic CBT interventions. MS participants have found generic CBT interventions less relevant to their needs [2]. A pilot study by Kiropoulos and colleagues [2] found that a tailored early CBT intervention compared to treatment as usual was effective for treating depressive symptoms amongst patients who were within 5 years of being diagnosed with MS. Large between-group treatment effects were found for level of depression at both post-intervention (*d* = 1.66) and 20 weeks follow-up (*d* = 1.34). Those in the tailored CBT intervention also reported significantly greater improvements in quality of life, fatigue and pain management, coping, and sleep quality and reductions in anxiety with medium to large effect sizes at post-intervention and at 20 weeks follow-up (*d* = 1.06–0.44).

### Rationale for the present study

Critique of previous research examining CBT for treatment of depression in those with MS includes lack of active comparator arms, small sample sizes, assessors and therapists not being blinded to participant allocation and a lack of research focused on newly diagnosed individuals [2, 22]. We have developed a tailored CBT intervention for depression and other MS-related concerns including anxiety, fatigue, pain and sleep disturbance for mild and moderately depressed individuals with MS who are within 5 years of being diagnosed. The current study will be a randomised controlled trial comparing the tailored CBT intervention with supportive listening (SL). This will allow us to assess whether there are any particular benefits associated with the tailored CBT intervention compared to SL.

### Objectives

The *primary aim* of this study is to assess the efficacy of a tailored early CBT intervention in treating depression (primary outcome) in individuals who have been newly diagnosed with MS and are experiencing mild to moderate depression. *Secondary aims* of the study are to examine whether those that undertake the CBT intervention will also display significant and clinically meaningful reductions in levels of anxiety. *Tertiary aims* of the study are to examine whether those that undertake the early tailored CBT intervention will also display significantly and clinically meaningful reductions in fatigue and pain impact, and significant improvements in sleep quality, MS-related quality of life, active coping styles, level of MS diagnosis acceptance, resilience and social support. *Quarternary aims* of the study are to examine the cost-effectiveness of the CBT intervention, taking into account benefits to patients, health service usage and cost of interventions.

### Hypotheses

Our *primary hypothesis* is that there will be a significantly higher percentage of participants who achieve a clinically meaningful change of 10 points or more on the Beck Depression Inventory-II (BDI-II) [23] between baseline and post in the CBT intervention compared to those receiving the SL intervention. Our *secondary hypotheses* are that those in the CBT intervention will display a significantly greater change in the BDI-II total score at 20 weeks follow-up compared to those in the SL intervention; and that there will be a significantly higher percentage of participants who achieve a clinically meaningful change of 10 points or more on the State Trait Anxiety Inventory (STAI) [24] between baseline and post in the CBT intervention compared to those receiving the SL intervention. Our *tertiary hypotheses* are that those in the CBT intervention will display a significantly greater change in the STAI total score at 20 weeks follow-up compared to those in the SL intervention; and that those in the CBT intervention will display significant reductions in fatigue and pain impact and significant improvements in level of MS illness acceptance, MS-related quality of life, sleep quality, active coping strategies and resilience at post-intervention (8 weeks) and at 20 weeks follow-up.

## Methods/design

**Fig. 1:**
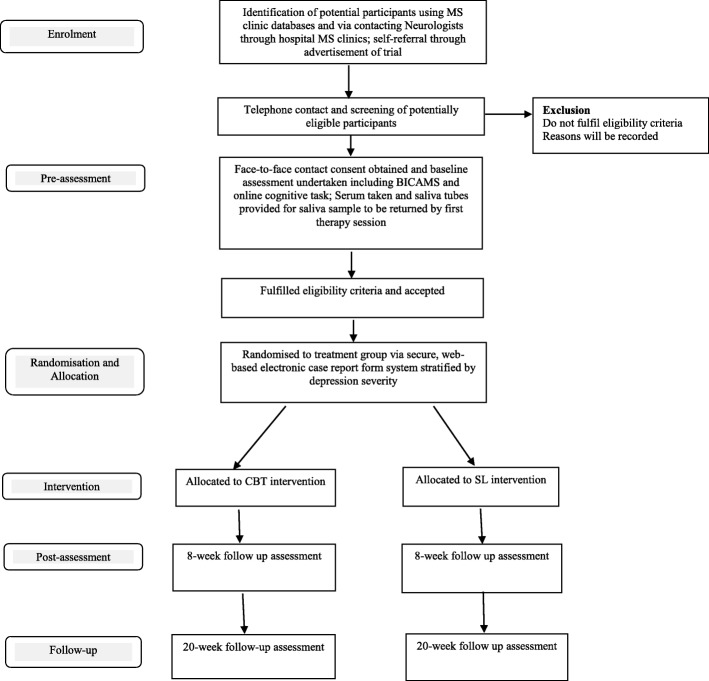
CONSORT flow chart of participants through the ACTION-MS trial

This protocol is reported according to guidelines presented in the Consolidated Standards of Reporting Trials (CONSORT) 2010 statement for clinical trial protocols [25]. Figure 1 displays the CONSORT flow chart of participants through the trial. The Standard Protocol Items: Recommendations for Interventional Trials (SPIRIT) checklist is provided as Additional file 1.

### Trial design

The ACTION-MS trial is a prospective, parallel group, assessor-blinded, active comparator, stratified, clinical trial with balanced randomisation (i.e. 1: 1).

### Participant recruitment

Participants will be recruited from MS clinics located at the Royal Melbourne Hospital (RMH), St Vincent’s Hospital and Western Health, Victoria, Australia. Treating neurologists, nurse co-ordinators and a researcher located at these clinics will screen patients with MS who are within 5 years of having received a diagnosis using a screening questionnaire asking two questions (Have you been feeling low most days for the last 2 weeks? and Have you lost interest or pleasure in most things?). If patients positively endorse at least one of these questions and they consent to being contacted, their contact details will be given to the trial manager for further assessment. The trial will be advertised through hospital and MS-related websites, and individuals can also self-refer to the study. The trial manager will review and confirm eligibility of participants. Individuals will be invited to an appointment for a baseline assessment at the RMH site. A total sample of 60 individuals newly diagnosed with MS will be enrolled to take part in the current study based on our pilot trial data [2].

### Consenting participants

Individuals will provide informed consent to participate when they attend the baseline assessment with a clinical psychologist or registrar. No study-specific procedures will take place before written consent has been obtained. After the baseline assessment, the clinical trial manager will confirm eligibility with the participant.

### Inclusion and exclusion criteria

Eligible participants will be a group of mild to moderately depressed patients (i.e. those who have scored between 14 and 28 on the BDI-II) who have been diagnosed with MS by a neurologist within the last 5 years. Exclusion criteria include (1) gross cognitive impairment that would make participation in the eight 1-h sessions of CBT distressing. Cognitive impairment will be assessed with the Brief International Cognitive Assessment for MS (BICAMS) [26]; (2) inability to speak or read English; (3) acute organic brain syndrome (e.g. delirium); (4) serious psychological disorder (e.g. psychosis); (5) those assessed with the BDI-II (a score of 28 or more) and the Structured Clinical Interview for DSM-5 Research Version (SCID-5-RV) [27] as being severely depressed; and (6) currently undertaking psychological treatment for depression and/or anxiety. Those who are taking anti-depressant medication will be accepted after being on the medication for 2 months or longer. Any changes to disease-modifying medication will be recorded.

### Assessments

Patients in both the CBT and SL groups are assessed at three time points: at baseline (T1), at the end of the intervention at 8 weeks (T2) and at 20 weeks follow-up (T3). Participants will remain part of the study for 5 months. The psychological assessment will include collection of demographic information (e.g. age, marital status, work, educational level), MS disease characteristics (e.g. disease-modifying medications, type of MS) and psychiatric history, completion of an online study questionnaire housed on a Research Electronic Data Capture (REDCap) online system on a secure server, administration of the BICAMS [26], completion of an online cognitive task, conduction of the SCID-5-RV [27] and Serum and Saliva collection. The treating psychologists and trial manager will be unblinded to the intervention. Assessments take may take up to 2 h to complete.

### Randomisation

Randomisation with permuted blocks of different sizes into the tailored CBT intervention and SL arms (1:1 ratio) will be undertaken via a secure, web-based Electronic Case Report Form (ECRF) system. The randomisation will be stratified by depression severity (mild and moderate depression classified using the BDI-II). Randomisation takes place after baseline assessment data has been collected. The randomisation table was created by an independent statistician and is concealed from study investigators and the assessment psychologists. Figure 1 shows the study CONSORT flow chart of participants in this trial. The trial manager will log into the ECRF system that will randomise participants into intervention group and will notify the treating psychologist about intervention allocation for the participant.

### Blinding

The clinical psychologist and registrar undertaking the baseline and outcome assessments will be blinded to participants’ treatment allocation.

### Interventions and treatment protocol

Participants will be randomised to either the CBT or the SL intervention. For both interventions, participants will attend weekly therapy sessions over an 8-week period. The first session will be 90 min long; the remainder sessions will be 60 min. All sessions will be face to face and will be undertaken in a consultation room in the RMH. Both interventions will be carried out in accordance with written and structured manuals. Participants will be issued with either a CBT or an SL client manual in their first session. The CBT and SL interventions will start within 1 week post-randomisation.

#### Tailored cognitive behavioural therapy

The aim of the tailored intervention is to significantly decrease levels of depression (primary outcome), anxiety (secondary outcome) and fatigue and pain impact and see improvements in levels of quality of life, fatigue and pain impact, sleep difficulties, MS illness acceptance, active coping skills, social support and resilience (tertiary outcomes). The tailored CBT intervention is derived from Beck’s cognitive theoretical model for the treatment of depression in adults [28]. Table 1 provides an overview of the modules included in the tailored CBT intervention.

**Table 1:** Overview of the eight modules included in the tailored CBT intervention

	Title and description	CBT strategies
Module 1	Introduction to the tailored CBT program	Psycho-education on CBT model for treatment of depression and anxiety, fatigue and pain management, sleep hygiene; the link between thoughts, emotions, and behaviour; self-assessment activity identifying depressive, anxiety and MS symptoms; introduction to grief and loss model and acceptance of MS illness; self- assessment of stage of grief/loss; introduction of thought monitoring forms and provision of psycho-educational reading materials on depression, anxiety and MS and adjusting to living with MS
Module 2 and Module 3	Managing depressive and anxiety symptoms	Assist client to identify link between trigger events, thoughts, emotions and behaviour; identify situations that trigger low mood and anxiety; introduction to unhelpful thinking styles and how they contribute and maintain low mood and anxiety; introduce and discuss behavioural activation strategies such as pleasant activity scheduling including physical activity; mindfulness-based controlled breathing; progressive muscle relaxation to assist in the management of negative emotions; provide guided controlled breathing, progressive muscle relaxation and sleep hygiene audio tracks; provide psycho-educational material on the benefits of controlled breathing, progressive muscle relaxation and sleep hygiene
Module 4	Challenging unhelpful thoughts	Identifying own unhelpful thinking styles/errors using the thought monitoring form; discussion of how unhelpful thinking styles lead to negative emotions and unhelpful behaviours; introduction of how to manage and challenge unhelpful thoughts; finding alternative thoughts activity in session; provide psycho-educational material about challenging negative thoughts and coming up with alternative more helpful thoughts; provide thought monitoring, relaxation and pleasant activities forms for homework
Module 5	Managing fatigue and pain	Understanding pain and fatigue in MS from a cognitive behavioural perspective; discussion of pain and fatigue “traps”; provide psycho-educational material on fatigue and pain and depression cycle; identification of individual fatigue and pain cycle; identification of negative thoughts; identifying unhelpful thinking styles related to fatigue and pain; identification and discussion of more helpful thoughts
Module 6	Sleep hygiene	What is good sleep hygiene? Discussion of strategies to improve sleep such as establishing good sleep habits; provide psycho-educational material around factors that influence sleep
Module 7	Problem solving	Introduction to problem solving; discussion of how to problem solve in session using a client’s example; provide psycho-educational reading material on problem solving
Module 8	Relapse prevention and preparing for the future	Recap on all homework exercises and strategies covered in the intervention; discussion around managing depressive and anxiety symptoms, fatigue, pain and sleep problems; identifying triggers and early warning signs of depression relapse; discussion of strategies that have helped client; completion of depression relapse prevention plan in session

#### Supportive listening

**Fig. 2:**
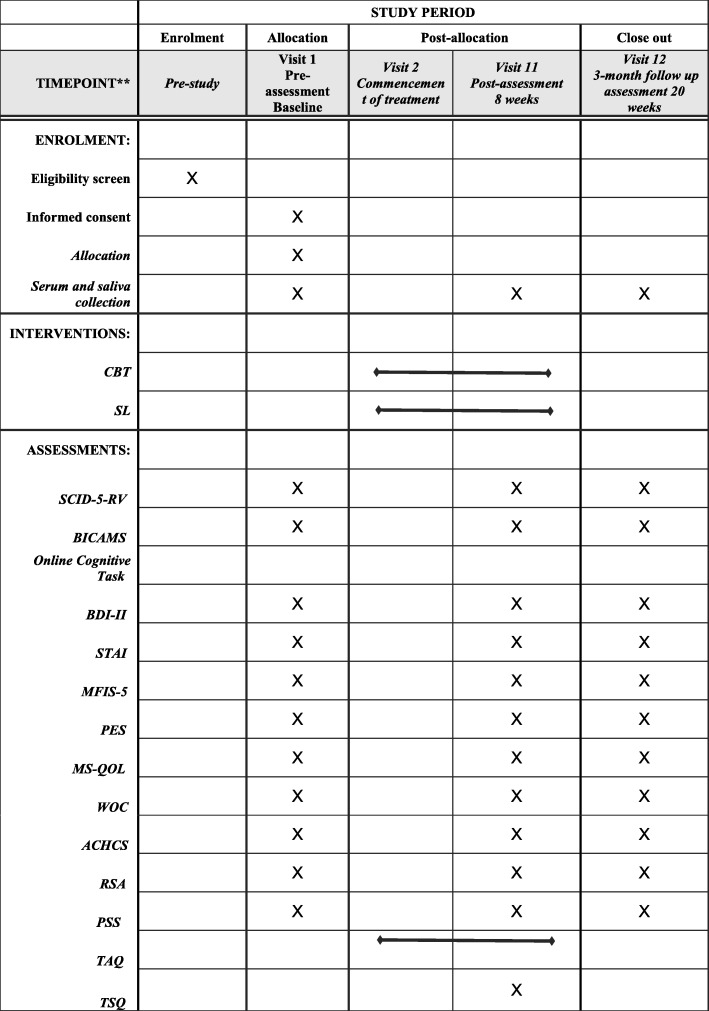
SPIRIT schedule of enrolment, interventions and assessments. *CBT* cognitive behavioural therapy, *SL* supportive listening, *SCID-5-RV* Structured Clinical Interview for DSM-5 Research Version, *BICAMS* Brief International Cognitive Assessment for MS, *BDI-II* Beck Depression Inventory-II, *STAI* State Trait Anxiety Inventory, *MFIS-5* Modified Fatigue Impact Scale-5 item version, *PES* Pain Effects Scale, *MS-QOL* multiple sclerosis-related quality of life measure, *WOC* 66 -item Ways of Coping questionnaire, *ACHCS* Acceptance of Chronic Health Conditions Scale, *RSA* Resilience Scale for Adults, *PSSS* Perceived Stress Scale, *TAQ* Therapeutic Alliance Questionnaire, *TSQ* Therapy Satisfaction Questionnaire

SL includes listening skills based on the theory and counselling technique of Carl Rogers [29]. Core listening skills such as paraphrasing, reflecting, summarising and asking open-ended questions are employed. The aim is to provide the participants the opportunity to talk and express themselves in a non-judgmental, safe environment in which they experience empathy from the therapist and feel listened to. Sessions in the SL group are not structured. Participants can choose topics to discuss with their psychologist which they think are currently relevant to them. Figure 2 displays the SPIRIT schedule of enrolment, interventions and assessments.

#### Training and supervision of therapists providing the interventions

Therapists will be registered clinical psychologists and registrars with training in CBT. All therapists will complete a 2-day workshop during which they will receive training in the two therapy techniques, with a day devoted to each of the CBT and SL interventions. All therapists will follow strict manualised protocols under the supervision of the trial manager.

#### Standardisation of the intervention

Standardisation of the intervention will be achieved by (1) use of a detailed intervention protocol which includes a therapist and client manual; (2) external monitoring of protocol compliance with routine feedback, through weekly supervision by the trial manager with the treating psychologists; (3) a protocol reminder system on our web-based data entry and management tool; and (4) a Data Safety and Monitoring Committee (DSMC) who will review protocol compliance. The DSMC will comprise an independent psychologist, neurologist and statistician who will be in contact with the principal researcher and trial manager.

#### Treatment fidelity

All intervention sessions will be recorded and used in supervision to provide feedback to therapists and to ensure treatment fidelity. We aim to take a 25% sample of intervention sessions for those in the CBT and SL groups from participants who agree to be recorded based on a random sample generated from an algorithm that takes into account session number, therapist and patient gender. Quality of CBT and SL administered will be assessed using the Cognitive Therapy Rating Scale [30] and a Supportive Listening Rating Scale. In addition, therapists in each condition will be asked to keep a self-reported fidelity checklist of all sessions.

### Outcome measures

#### Primary outcome measure

The primary outcome is the percentage of participants achieving the clinically meaningful change of 10 points or more on the BDI-II between baseline and post-intervention in the CBT intervention. Level of depression will be measured with the BDI-II.

#### Secondary outcome measures

Secondary outcomes are the magnitude of change between baseline and 20 weeks on the BDI-II in the CBT intervention with the level of depression measured with the BDI-II and the magnitude of change between baseline and post-intervention on the STAI. Level of anxiety will be measured with the STAI.

#### Tertiary outcome measures

Tertiary outcomes are the magnitude of change between baseline and post-intervention on fatigue and pain impact, MS-related quality of life, sleep quality, MS diagnosis acceptance, active coping styles, resilience and perceived social support in the early CBT intervention. Fatigue impact will be measured with the Modified Fatigue Impact Scale-5 item version (MFIS-5) [31]. Pain impact will be measured using the 6-item Pain Effects Scale [31]. Sleep quality will be measured with the Pittsburgh Sleep Quality Index [32]. MS-related quality of life is measured using the 54-item MS-related quality of life measure [33]. Coping styles will be examined using the 66-item Ways of Coping questionnaire [34]. Acceptance of MS illness will be measured using the 10-item Acceptance of Chronic Health Conditions Scale [35]. Level of resilience will be measured with the 33-item Resilience Scale for Adults [36], and Perceived Social Support will be measured using the 12-item Scale of Perceived Social Support [37].

#### Process measures

The Therapeutic Alliance Questionnaire [38] will be given to participants at each session, and they will be asked to complete and place it in an envelope which will be given to the therapist the following week. At post-intervention we will ask participants to complete a Therapy Satisfaction Questionnaire to provide information about the acceptability of the CBT intervention.

#### Saliva and serum samples

Participants will be asked to provide serum samples at baseline, post-intervention (8 weeks) and at 20 weeks follow-up. Additional consent for their use in future related research will be obtained.

#### BICAMS

We will collect test-retest reliability data for BICAMS from all participants enrolled in the trial. Participants will complete the BICAMS at the baseline assessment and again 1 week after this and prior to commencing their first therapy session. Additional consent for this data to be used in future related research will be obtained.

#### Sample size calculation

The current study is powered for the primary outcome of the proportion of patients who achieve a clinically significant change of 10 points or more on the BDI-II between baseline and post-treatment in the CBT intervention compared to those receiving the SL intervention. Based on pilot data by Kiropoulos and colleagues [2], the current study will aim to recruit 30 participants in each treatment arm (CBT and SL). The pilot results also revealed that 33% of newly diagnosed individuals screened did not meet eligibility to participate in the trial. Therefore, the current study will aim to screen 90 patients newly diagnosed with MS across three MS clinics in three hospital sites and via advertisements in order to obtain a total sample size of 60. This number will yield 90% power using an α = 0.05 threshold to observe a conservatively estimated 45% difference in proportion of patients achieving a favourable primary outcome. The sample size has also been inflated for drop-outs throughout the 20-week follow-up study.

### Planned statistical analysis

#### Effectiveness analysis

The primary outcome analysis will be an intention-to-treat between-group comparison of the proportion of patients who achieve a clinical and meaningful change of 10 points or more on the BDI-II between baseline and post-intervention adjusted for baseline depression severity using a logistic regression model. Baseline characteristics will be presented by randomised group without formal statistical tests. Secondary and tertiary outcome analyses will be undertaken using appropriate regression models adjusted for baseline depression severity. Longitudinal changes will be examined using regression models. No formal interim analyses for either efficacy or safety are planned, but safety outcomes will be continuously monitored.

#### Health economics evaluation

Participants will be asked to record service use including all types and duration of hospital admissions, frequency of outpatient hospital appointments, MS-related community service use, anti-depressant and anti-anxiety medication use and cost and paid and unpaid work using the Client Service Receipt Inventory [39]. Health-related quality of life data and quality-adjusted life years (QALYs) will be assessed using the health-related quality of life measure (EuroQoL-5D) [40].

#### Trial monitoring and management and patient safety

The study will be co-ordinated by a trial manager who will be a clinical psychologist. Trial monitoring and management will be undertaken by (1) a steering committee who will meet once a year to discuss the overall conduct of the trial and will include the lead investigator and key investigators from the recruitment sites, the trial manager and the statistician; (2) a management committee who will meet fortnightly to monthly to discuss recruitment issues and will include the trial manager and the key investigators from the hospital sites; and (3) safety monitoring, as performed by the independent DSMC who will provide advice regarding the safety of the trial to the steering committee and will consist of a clinician, a psychologist and an independent statistician. We will continuously monitor serious adverse events. All adverse events will be prospectively classified as serious or not serious. An independent clinical adjudication committee adjudicates all serious adverse events for this trial for subsequent reporting to the DSMC. In the unlikely case of an adverse event, this will be documented by the principal researcher and trial manager. Precautions have been taken to reduce the likelihood of adverse events occurring such as the exclusion of patients who are acutely suicidal or severely depressed. The interventions are delivered by clinical psychologists or registrars under the weekly clinical supervision of the trial manager. In the case of any adverse events as a result of taking part in either of the interventions, participation in the trial will be discontinued. Serious adverse events will be reported to the ethics committee. Any adverse events will be reported in the trial paper.

#### End of study

The end of the study is defined as the last data collection during the last 20 weeks follow-up visit from the last participant after the CBT or SL intervention, whichever comes last.

#### Data management, protection, storage and archiving of study documents

Access to participant data will be restricted to the principal researcher and appropriate study staff as needed. All laboratory specimens and questionnaire forms will be identified using unique participant ID numbers to maintain participant confidentiality. All records including the serum and saliva samples will be kept in secure storage areas with limited access to study staff only. Personal data and anonymised data files will be stored in locked filing cabinets for hard copies and in a secure online database housed on the University of Melbourne secured server with access via password-protected computers by study staff only. All study documentation will be kept for a minimum of 5 years from the protocol-defined end-of-study point in the University of Melbourne archive. All documentation will be destroyed after this date.

#### Participant adherence

Adherence to the CBT intervention will be defined as attendance of the baseline assessment session and attendance and completion of at least four CBT sessions with a psychologist. We will also monitor patient adherence to the CBT intervention through completion of a patient satisfaction questionnaire at the 8 weeks post-assessment. Participant drop-out and reason will be recorded.

#### Withdrawal procedures

Participants will have the option to withdraw at any time during the study period of 5 months. The participant will need to request this formally, and the reason for withdrawing from the study will be recorded by the trial manager. Participant data collected prior to the withdrawal will be used with the permission of the participant. A participant can be withdrawn by the chief investigator should the need arise throughout the study period, and this will also be recorded.

#### Patient and public involvement

Patients have been involved in the development of the research question and the prioritisation of depression and related factors in the newly diagnosed period as a subject for research. In our pilot trial, patients were involved in a semi-structured qualitative interview where they gave feedback about the content of the tailored CBT intervention and their experience of participating in the trial using the tailored CBT intervention manual [2]. Patients provided feedback about the feasibility, usefulness, appropriateness and content of the tailored CBT intervention [2]. We will send all participants a report describing the findings and their implications.

## Discussion

Depression is common in individuals newly diagnosed with MS [1, 2] and, if left untreated, it will contribute to further deterioration, having an impact on the course of MS [19] and immune functioning [5], result in the exacerbation of MS relapses [17], contribute to higher suicide rates [20] and possibly affect adherence to medical advice and treatments [16, 21]. There is a scarcity of psychological interventions for depression targeting the newly diagnosed period. Such interventions have the potential to have a major impact on the mental and physical wellbeing of those newly diagnosed with MS [2]. Our pilot trial data has shown the tailored CBT intervention to significantly reduce depressive and anxiety symptoms and provide an increase in quality of life and better management of fatigue, pain and sleep disturbances [2] in those newly diagnosed with MS. The current trial will test the efficacy of a tailored CBT intervention for depression. It will provide a range of effect size estimates that can be used to power a later-stage multi-centre trial of treatment efficacy, it will inform the mechanisms underlying any treatment effects and will provide acceptability and cost-effectiveness data for delivering this intervention through outpatient MS clinics.

### Trial status

The ACTION-MS trial began recruitment on 14 June 2017 using protocol version number 1, 14 June 2016. It is anticipated that recruitment will be ongoing until December 2021. It is expected that data collection will be completed by April 2022.

## Supplementary information

**Additional file 1.** SPIRIT 2013 checklist: recommended items to address in a clinical trial protocol and related documents.

## Data Availability

The datasets used and/or analysed during the current study are available from the corresponding author on reasonable request.

## Abbreviations

BDI-II: Beck Depression Inventory-II
BICAMS: Brief International Cognitive Assessment for MS
CBT: Cognitive behavioural therapy
CONSORT: Consolidated Standards of Reporting Trials
DSMC: Data Safety and Monitoring Committee
ECRF: Electronic Case Report Form
MS: Multiple sclerosis
QALY: Quality adjusted life year
RMH: Royal Melbourne Hospital
SCID-5-RV: Structured Clinical Interview for DSM-5 Research Version
SL: Supportive listening
SPIRIT: Standard Protocol Items: Recommendations for Interventional Trials
STAI: State Trait Anxiety Inventory
