# Correction to: Comparison of the effectiveness of a tailored cognitive behavioural therapy with a supportive listening intervention for depression in those newly diagnosed with multiple sclerosis (the ACTION-MS trial): protocol of an assessor-blinded, active comparator, randomised controlled trial

**DOI:** 10.1186/s13063-020-04261-x

**Published:** 2020-03-20

**Authors:** Litza Kiropoulos, Trevor Kilpatrick, Tomas Kalincik, Leonid Churilov, Elizabeth McDonald, Tissa Wijeratne, Jennifer Threader, Vanja Rozenblat, Neil O’Brien-Simpson, Anneke Van Der Walt, Lisa Taylor

**Affiliations:** 1 Melbourne School of Psychological Sciences, University of Melbourne, Melbourne, Victoria 3010 Australia; 2 Department of Neurology, Royal Melbourne Hospital, Melbourne, Victoria Australia; 3 Florey Institute of Neuroscience and Mental Health, Melbourne, Victoria Australia; 4 Department of Neuroscience, Rehabilitation and Neuroimmunology, St Vincent’s Hospital, Melbourne, Victoria Australia; 5 Department of Neurology, Western Health, Sunshine, Victoria Australia; 6 Melbourne Dental School, University of Melbourne, Carlton, Victoria Australia; 7 Department of Neurosciences, Monash University, Melbourne, Victoria Australia

**Correction to: Trials (2020) 21: 100**

**https://doi.org/10.1186/s13063-019-4018-8**

After publication of our article [1] the authors have notified us that three of the names have been incorrectly spelled.

- Original name spelling:
  Tomas Kalincek
  Leonid Cherulov
  Neil Simpson-O’Brien
- Correct name spelling:
  Tomas Kalincik
  Leonid Churilov
  Neil O’Brien-Simpson
